# Portuguese parental beliefs and attitudes towards vaccination

**DOI:** 10.1080/21642850.2021.1920948

**Published:** 2021-05-06

**Authors:** Inês C. Fonseca, Ana Isabel Pereira, Luísa Barros

**Affiliations:** a Faculdade de Psicologia, Universidade de Lisboa, Alameda da Universidade, Lisboa, Portugal; b CICPsi, Centro de Investigação em Psicologia, Universidade de Lisboa Alameda da Universidade, Lisboa, Portugal

**Keywords:** Pediatric vaccination, intention, parental beliefs, vaccine confidence, vaccine complacency

## Abstract

**Introduction:**

Portugal has one of the highest vaccine coverage rates among European countries, associated with excellent vaccine convenience and confidence levels. Considering both the high rate of pediatric vaccination in Portugal and the excellent indicators of vaccine convenience established, an analysis of confidence and complacency indicators could help understand this positive example. This study aimed to characterize parental beliefs according to the intention to vaccinate a next child and identify cognitive and demographic predictors of that intention in a Portuguese sample.

**Methods:**

We measured perceptions of vaccines’ safety and efficacy, perceptions of the severity of vaccine-preventable diseases, beliefs related to conspiracy theories, attitudes towards immunization requirements, perceptions of social norms as predictors of the intention to vaccinate a subsequent child. We also inquired if parents had previously refused a recommended vaccine. The authors disseminated the questionnaire online to reach a diverse population of parents of 0–12 years old children. The final sample included 1,118 parents, 96.9% reported their intention to vaccinate the next child, and 3.6% had previously refused a vaccine. Two additional open-ended questions regarding motives to vaccinate or refuse a future baby's vaccination were answered by 886 parents.

**Results:**

All the evaluated parental cognitive dimensions were significantly different between the group of parents who would vaccinate a next child and those who expressed the intention not to vaccinate. Beliefs about the safety and efficacy of vaccines and having fewer children were significant predictors of that intention.

**Conclusion:**

The vast majority of parents reported attitudes and beliefs favorable to pediatric vaccination with high consistency in all cognitive dimensions assessed. Concerns regarding pediatric vaccines’ safety need to be sensitively and actively addressed by health providers to maintain excellent vaccination coverage rates.

Despite the significant advances in child health due to the generalization of pediatric immunization during the twentieth century, many Western countries have seen decreased rates of childhood vaccination and a rise in anti-vaccine movements in the last decade, with dramatic consequences for the spread of infectious diseases (Larson, Jarrett, Eckersberger, Smith, & Paterson, 2014; Larson et al., 2016; Larson, Figueiredo, Karafillakis, & Rawal, 2018).

Determinants of vaccination rates are multiple and complex. The SAGE (synthetic, augmentative, generative, experiential) model of vaccine hesitancy determinants (World Health Organization [WHO], 2014) proposes three groups of factors to explain vaccine adherence or refusal, namely, (a) convenience (physical availability, affordability, and accessibility); (b) complacency (low perceived risks of vaccine-preventable diseases leading to the vaccination being deemed unnecessary) and (c) confidence (trust in the safety and efficacy of the vaccines, the health system and the motivations of policymakers who select a vaccine).

In the context of multiple threats to pediatric immunization programs, Portugal stands out with one of the highest rates of pediatric vaccine coverage in Europe. According to the State of Vaccine Confidence in the European Union (EU) 2018 study, Portugal has the highest percentage of respondents agreeing that vaccines are safe (95.1%), effective (96.6%), and important for children (98.0%) (Larson et al., 2016). While measles coverage and global vaccine confidence have declined in several EU member states, they have remained stable and over 95% in Portugal since 2000 (Direção Geral de Saúde [DGS], 2019). Human Papiloma Virus (HPV) immunization coverage for girls at 14 years old has attained similar results (94%) (DGS, 2019). In 1965, Portugal implemented the National Immunization Program (NIP) with universal, state-funded, and free vaccines for all children living in the country, delivered through health centers, hospitals, and clinics (DGS, 2019). This program assures that the recommended pediatric vaccines are readily available, accessible, and affordable for all families. The NIP includes 11 vaccines for children up to 18 years old: hepatitis B, Haemophilus influenza type b, diphtheria, tetanus and pertussis, poliovirus vaccine, Streptococcus pneumoniae, Neisseria meningitis C, and measles, mumps, and rubella vaccines. And for ten years old girls, the HPV vaccine. Official guidelines for health services define that all contact with these services should be used to complete or update the immunization scheme (DGS, 2019). Although vaccines are not mandatory, there is a comprehensive monitoring system, including checking for vaccine compliance by healthcare centers, daycares, schools, summer camps, and other child institutions. Together, these conditions ensure very favorable conditions for pediatric immunization with high levels of vaccine convenience.

Based on the SAGE framework and considering both the high rate of pediatric vaccination in Portugal and the excellent conditions for vaccine convenience described above, analyzing confidence and complacency indicators could help to understand this positive example. Portuguese parents are not protected from the spread of anti-vaccination rhetoric (Smith & Graham, 2019). They are frequent users of parenting blogs and other online resources and use these to access information about child health; besides assessing Portuguese-speaking websites, most of them also use English language sources (Santos, Gago, Perdomo, & Rodrigo, 2018). Additionally, the high rates of vaccine coverage might contribute to a decrease in the perception of risks associated with vaccine-preventable diseases and, as such, increase complacency-related beliefs (Kennedy, Basket, & Sheedy, 2011; Harmsen et al., 2012a).

According to the protection motivation theory (Rogers,^1975^), a high perception of the severity of vaccine-preventable diseases (threat appraisal) and a high perception of the safety and efficacy of the recommended vaccines (coping appraisal) would determine the protection motivation, i.e. the intention to engage in the behavior. The theory of planned behavior (Azjen, 1991) also emphasizes the role of social norms, i.e. the perception that other relevant people would approve our own engagement in a specific behavior. More recently, exposure to anti-vaccine conspiracy theories, i.e. beliefs about powerful and manipulative forces, such as large pharmaceutical companies and governments imposing immunization recommendations to meet economic or political objectives, has been associated with a reduced overall intention to vaccinate (Jolley & Douglas, 2014). Systematic literature reviews have identified different parental cognitions associated with vaccine hesitancy and refusals, such as negative beliefs regarding the safety and efficacy of pediatric vaccines, the low perception of the severity of vaccine-preventable diseases, the social norms regarding the vaccination of children, and the belief in conspiracy theories related to recommended vaccine programs (Martins et al., 2015; Smith et al., 2011). It is probable that many of these beliefs occur concomitantly in the same person and may even reinforce one another. However, most of the existing studies do not consider all these cognitive dimensions simultaneously, thus not allowing to assess what cognitions are significantly and independently associated with a positive intention to vaccinate.

Beyond cognitive predictors, previous studies have shown that sociodemographic variables are associated with a higher probability of people accepting anti-vaccination ideas and refusing to vaccinate their children (Salmon et al., 2005). However, there are still knowledge gaps in this field. In particular, the results regarding education level are often contradictory (Larson et al., 2014). Additionally, the association of vaccine hesitancy with the family dimension and structure is still unclear (Vandermeulen, Roelants, Theeten, Van Damme, & Hoppenbrouwers, 2008).

Considering the specific Portuguese situation within the framework of the SAGE model and the excellent convenience conditions for pediatric vaccination, this cross-sectional study explored the dimensions of pediatric confidence and complacency beliefs in a sample of Portuguese parents, according to their intention to vaccinate a next child with the nationally recommended vaccines. Intention to vaccinate a potential new baby was previously associated with higher levels of fully immunized children (Gust et al., 2004). Specifically, we aimed to identify the following: 1) beliefs regarding vaccine safety and efficacy, social norms regarding pediatric vaccination, and the belief in conspiracy theories (confidence); 2) beliefs regarding the perceived severity of vaccine-preventable diseases and the acceptance of vaccine requirements (complacency); and 3) the predictors of parents’ pediatric vaccine intention considering the combined effects of sociodemographic and cognitive determinants. In this report, we included all the vaccines recommended by the NIP for pediatric ages. We expected to find very high rates of vaccine-favorable beliefs and an association between positive beliefs and the intention to vaccinate the next child.

## Methods

### Participants

Parents or guardians of at least one 0-12-year-old child were eligible to participate. The final sample included 1,118 individuals aged 18–58 years old (*M* = 35.28; *SD* = 0.17); 94.5% were mothers, 72.9% had post-secondary education, and lived in the 20 different districts of mainland Portugal and the two archipelagos. A subgroup of 886 participants agreed to answer two optional open questions: 21–52 years old (*M* = 36.2; *SD* = 5.26), 94.2% mothers, and 78.3% completed post-secondary studies. From these, 860 reported they would vaccinate a next child, and 26 would not Table 1.

**Table 1.** Sociodemographic data of the total sample (N = 1118) and the subsample who answered optional questions (N = 886).

	*N*	%	M (SD)
Total sample			
Age	–	–	35.3 (0.17)
Mothers	1057	94.5%	–
Fathers	61	5.5%	–
*Level of Education*			
Elementary Education (9 years)	56	5.0%	–
Secondary Education (12 years)	539	22.1%	–
Post-secondary	815	72.9%	–
*Number of Children*			
One Child	627	56.1%	–
More Than One Child	491	43.9%	–
*Youngest Child's age*			
0–2 years old	548	49.0%	–
> 2 years old	570	51.0%	–
*Subsample open-ended questions*	886	79.2%	–
Age	–	–	36.2 (5.26)
Mothers	835	94.2%	–
Fathers	51	5.8%	–
*Level of Education*			
Elementary Education (9 years)	20	2.3%	–
Secondary Education (12 years)	172	19.4%	–
Post-secondary	694	78.3%	–

### Measures

*A sociodemographic questionnaire* included questions regarding participants’ age, gender, educational level, region of residence, occupation, and children's number and age.

*Intention to vaccinate* was assessed with a question similar to the one in the Parent Attitudes about Childhood Vaccines scale (Gust et al., 2004) ("If you had another baby today, would you want him/her to get all recommended immunizations?") with a dichotomous answer (yes or no). We used a forced dichotomous choice, similar to previous studies (Harmsen et al., 2012b; Yeh, 2015), as we expected a very biased distribution in the intention to vaccinate.

*Previous refusal to vaccinate.* A single dichotomous question was used to evaluate previous refusal to vaccinate ("Have you ever refused to vaccinate your child with a vaccinate from the NIP") (Gilkey, Calo, Marciniak, & Brewer, 2017).

*Vaccine Safety and Efficacy and Severity of Vaccine-Preventable Diseases*. To develop this measure, we selected items translated and adapted from previous questionnaires (Gust et al., 2004; Larson et al., 2015; Salmon et al., 2005; Shapiro et al., 2018; Smith et al., 2011; Stefanoff et al., 2010). We gathered a pool of 20 items regarding the domains of vaccine safety (6 items), vaccine efficacy (9 items), and the severity of vaccine-preventable diseases (5 items). We selected four items for each scale based on the item distribution and internal consistency analysis to obtain a shorter version. The 12 items selected showed a less skewed and more balanced distribution and contributed to a higher internal consistency of the scale. Participants answered on a *Likert* scale from 1 (strongly disagree) to 5 (strongly agree) and items were inverted when necessary, so that higher values more favorable beliefs and attitudes towards vaccines. We conducted a principal component analysis on the 12 items. The Kaiser-Meyer-Olkin (KMO) was .94, supporting sample adequacy for the analysis, and Bartlett's test of sphericity (χ2(66) = 6198.51; *p* < 0.001) indicated that correlations between the items were large enough to conduct the analysis. Considering the Guttman-Kaiser decision criteria (eigenvalue > 1,0), two components, explaining a total of 58.3% of the variance, were extracted: safety and efficacy of vaccines (6 items – "Childhood vaccines are effective"; "Vaccines are safe"; "Children get more shots than are good for them"), and the severity of vaccine-preventable diseases (6 items – "I believe that many of the diseases that vaccines prevent are serious"; "Vaccination is important to protect the entire community from disease"). The factor loadings of the items after a Varimax rotation were between .74 and .55. Both subscales showed good internal consistency (safety and efficacy α = 0.82 and perceived severity α = 0.84).

*Conspiracy Beliefs* were assessed through the Anti-Vaccine Conspiracy Theories Scale (Jolley & Douglas, 2014) that targets participants’ agreement with ideas conveyed by the conspiracy theories about vaccination most disseminated by the media and social networks ("People are deceived about vaccine's effectiveness" and "Vaccines are harmful, and this fact is covered up"). In this study, the results showed good internal consistency (α = 0.86), in line with the original version. Participants answered on a *Likert* scale from 1 (strongly disagree) to 5 (strongly agree); to have all scales in the same direction items were inverted so that higher values represented more favorable attitudes towards vaccines.

*Acceptance of Vaccines Requirements* was assessed through The Vaccination Requirements Attitudes Scale (Salmon et al., 2005). This scale, with three items, was used to assess parents’ attitudes towards compliance with the NIP and school vaccination requirements (e.g. "I am opposed to school immunization requirements because parents know what is best for their children" and "Parents should be allowed to send their children to school even if their child is not vaccinated"). Participants answered on a *Likert* scale from 1 (strongly disagree) to 5 (strongly agree) and items were inverted when necessary, so that higher values more favorable attitudes towards vaccines. The internal consistency was good (α = 0.76).

*Social Norms* were assessed with the Perception of Social Norms About Pediatric Vaccination Scale (Coniglio, Platania, Privitera, Giammanco, & Pignato, 2011) that evaluates social norms regarding pediatric vaccination. Taking into account that the scale only had three items, the alpha of .63 was considered acceptable. ("The health professional I trust believes that I should vaccinate my children", "The people important to me (family or friends), believe that I should vaccinate my children", and "The people important to me (family and/or friends), believe that I should not vaccinate my children"). The interitem mean of .38 also supported the scale's internal consistency (Ponterotto & Ruckdeschel, 2007). Participants answered on a *Likert* scale from 1 (strongly disagree) to 5 (strongly agree) and items were inverted when necessary, so that higher values represented more favorable attitudes and beliefs towards vaccines.

*Motives to vaccinate and not vaccinate a child.* Finally, the participants responded to two optional open-ended questions adapted from Larson et al. (2015). Participants were asked about their reasons for vaccinating a child ("What are the three main reasons why you should vaccinate your child?") and to list the reasons that could lead some parents to refuse pediatric vaccination ("In your opinion, why do some people refuse the recommended vaccines for their children?").

All the scales and the open-ended questions were adapted for Portuguese through a forward–backward translation process according to recommended guidelines for test translation and adaptation (Muñiz, Elosua, & Hambleton, 2013): a) two independent translations of the original instrument, b) a conciliated version, c) a back translation by an independent, native English speaker translator, d) comparison of the original instrument with the new English translation by another independent, native English speaker translator, to assess equivalency of the semantics, e) pilot testing of the final version with five parents of children with 2- to 12-year-olds, to confirm the clarity of the items.

### Procedures

To recruit a diverse sample of participants, we invited parents to participate in an online study conducted during January 2019. Potential participants received an invitation via diverse social networks related to parenting, briefly explaining the study's objectives and conditions and including a link to access the questionnaires, supported by the Qualtrics software. Parents were also encouraged to invite other parents from their networks to participate. When accessing the platform, participants would find a brief presentation of the study objectives, the expected time for completion (approximately 5 min), and an informed consent form ensuring the anonymity and safeguarding of the collected information. This study obtained approval from the Ethics Committee of School of Psychology, University of Lisbon.

### Data analysis

We conducted a preliminary analysis of the item distribution, Cronbach's alpha, and the interitem correlation for all scales. After calculating the descriptive statistics of all scales, we analyzed the correlations between the variables. We used Chi-Squared test to explore the association between the previous refusal of a vaccine and the intention to vaccinate the next child. To analyze the differences among groups (according to the intention to vaccinate) regarding parental beliefs, t-tests for independent samples and Pearson's chi-squared tests were used. To compare intentions and beliefs according to educational level, we considered only two groups (elementary and secondary versus post-secondary education). Finally, after confirming the independent variables’ noncollinearity, a binary logistic regression considering the intention to vaccinate the next child as the dependent variable was performed.

We performed content analysis to identify the main categories of content in the answers to the two open-ended questions. Then, relative frequencies were calculated for the categories identified in each of the two groups of parents according to the intention to vaccinate the next child.

## Results

### Intention to vaccinate and previous refusal to vaccinate

The large majority of the participants expressed the intention to vaccinate a subsequent child. Only 3.1% (*N* = 35) reported an intention not to vaccinate, and 3.6% (*N* = 40) reported a previous refusal of a vaccine. Intention to vaccinate was not significantly different between parents with different education levels. However, parents with only one child (χ2 (1) = 4.98, *p* < .001) and parents with a child younger than two years old (χ2 (1) = 6.04, *p* < .05) were significantly more likely to vaccinate the next child. There was a significant negative association between the intention to vaccinate the next child and the refusal of a previous pediatric vaccine (χ2 (1) = 756.59, *p* < .001, *Φ* = -.82).

### Vaccine Safety and Efficacy and Severity of Vaccine-Preventable diseases

All cognitive dimensions evaluated were highly favorable for pediatric vaccination (*M* > 4), although the answers covered all Likert scale ranges. We found moderate to strong positive correlations between all subscales (*r* = .43 - .76) (Table 2), with the highest correlations found between safety and efficacy perception and conspiracy theories beliefs.

**Table 2.** Pearson's correlations between attitudes and beliefs scales; Descriptives about parental beliefs – mean (standard deviation), observed minimum and observed maximum (N = 1118).

	1.	2.	3.	4.	5.
1. Safety & Efficacy					
2. Severity of Diseases	.70**				
3. Acceptance of Vaccines Requirements	.64**	.65**			
4. Social Norm	.52**	.56**	.48**		
5. Conspiracy Beliefs	.76**	.60**	.59**	.43**	
Mean (SD)	4.25 (.02)	4.72 (.02)	4.45 (.02)	4.75 (.02)	4.08 (.03)
Observed Max	5	5	5	5	5
Observed Min	1	1	1	1	1

Note. A higher score on each scale indicates more favorable beliefs towards pediatric vaccination. ** *p* < .01.

### Beliefs according to intention to vaccinate

Parents who expressed the intention to vaccinate a next child expressed beliefs significantly more favorable to pediatric vaccines in all dimensions (Table 3), with large effects for all the analyses (Cohen *d* = 1.76–2.93).

**Table 3.** t-tests for the differences between groups with positive and negative intentions to vaccinate a next child (n No = 35, n Yes = 1083, and n Total = 1118).

	Intention to vaccinate a next child	*M (SD)*	t (df)
Safety & Efficacy	No Yes	1.87 (1.02) 4.32 (.60)	t (34.75) = −14.15***
Severity of Diseases	No Yes	2.80 (1.20) 4.78 (.36)	t (34.20) = −9.76***
Acceptance of Vaccines Requirements	No Yes	2.06 (1.07) 4.52 (.70)	t (34.42) = −8.03***
Social Norms	No Yes	3.42 (1.01) 4.79 (.44)	t (34.94) = −13.49**
Conspiracy Beliefs	No Yes	2.05 (1.11) 4.15 (.77)	t (35.07) = −11.06**

Note. A higher score in each scale indicates more favorable beliefs towards pediatric vaccination.

** *p* < .01. *** *p* < .001.

### Demographic and cognitive predictors of intention to vaccinate

To assess the conjoint effect of demographic and cognitive dimensions in predicting the intention to vaccinate a subsequent child, we conducted a binary logistic regression. We included all the beliefs related to pediatric vaccines and the sociodemographic variables that were significantly different in the two groups according to the intention to vaccinate as possible predictors (Table 4).

**Table 4.** Binary logistic regression, with the dependent variable being the intention to vaccinate a next child.

	B	SE	Wald	EXP (B)
Number of Children	−.82*	.40	4.11	0.44
Age of Youngest Child	−.07	.62	0.01	1.07
Safety & Efficacy	2.11**	.74	8.25	8.28
Severity of Diseases	.91	.57	2.52	2.48
*Acceptance of Vaccines Requirements*	.73	.42	3.21	2.07
Social Norms	.74	.46	2.60	2.10
Conspiracy Beliefs	−.89	.69	1.69	0.41
Model χ ^2^(1) = 24.14				

*p < .05; ** *p* < .01.

After adjusting for the effects of other variables in the model, positive beliefs about vaccines’ safety and efficacy and having fewer children were significant predictors of the intention to vaccinate a subsequent child.

## Qualitative data results

### Motives to vaccinate a child

When parents were asked about their motives to vaccinate a child in general, the main reasons identified by both parents who intended to vaccinate a next child (nIV = 860) and those who did not (nINV = 26) were to achieve individual (nIV = 852, 99.07%; nINV = 11, 42.3%) and community immunization (nIV = 400, 46.51%; nINV = 8, 30.76%). Only 3 (11.53%) of those who would not vaccinate a next child did not identify any reason to do so.

### Reasons why some parents refuse to vaccinate their children

Parents with a positive intention to vaccinate identified as reasons why some people do not vaccinate their children. These reasons included the parents’ lack of information or perception of a lack of rigorous research (*n* = 560, 65.12%); holding beliefs compatible with conspiracy theories like political ideologies, religious faith and trusting fake news (*n* = 255, 29.65%); the fear of possible longtime negative consequences (*n* = 157, 18.26%); a low perception of vaccine efficacy (*n* = 27, 3.14%) and irresponsibility (*n* = 42, 4.88%). A small percentage were incapable of identifying any reason (n = 25, 2.91%).

On the other hand, parents who expressed an intention not to vaccinate identified the fear of negative consequences (*n* = 15, 57.69%), followed by several beliefs typical of anti-vaccines conspiracy theories (*n* = 10, 38.46%), the perception of low efficacy of vaccines (*n* = 9, 34.62%), and a lack of research and information (*n* = 8, 30.77%).

## Discussion

This study reinforces previous reports about the highly positive attitudes, and beliefs towards pediatric vaccines (Gellin, Maibach, & Marcuse, 2000; Larson et al., 2018) observed in Portugal. Confirming several previous studies (Enkel, Attwell, Snelling, & Christian, 2018; Salmon et al., 2008; Smith et al., 2011), we also found that the differences in the intention of parents to vaccinate if they would have a new baby were associated with different levels of agreement with favorable beliefs towards pediatric vaccination. Parents who expressed the intention to vaccinate a subsequent child expressed more positive beliefs about vaccination in all evaluated dimensions, and this intention was negatively associated with a previous vaccine refusal.

Notwithstanding, we need to be careful in interpreting these results as the cross-sectional design does not allow us to infer a causal effect of these beliefs. Some parents may vaccinate their children simply because they follow medical advice, considering compliance with these guidelines as the natural and easiest way to proceed (Forster et al., 2016). However, we should highlight the consistency between all beliefs assessed and between beliefs and the intention to vaccinate. Parents who showed less concern about safety and efficacy or higher perception of the severity of vaccine-preventable diseases also endorsed more positive social norms regarding vaccines or were less willing to agree with anti-vaccines conspiracy theories. Salmon et al. (2008) found that the parents of unvaccinated children were more likely to report a low perception of the susceptibility and severity of vaccine-preventable diseases, a low perceived efficacy and safety of vaccines, and a low level of trust in government. Vaccine confidence depends not only on trusting vaccines but also on the overall trust in the system that produces them (Jamison, Quinn, & Freimuth, 2019).

According to the protection motivation model (Rogers, 1975) and the theory of planned behavior (Azjen, 1991), higher perceptions of disease severity and vaccine efficacy should predict higher intentions to vaccinate. In our study, when considering all relevant dimensions conjointly, positive beliefs about vaccines’ safety and efficacy was the only cognitive dimension that predicted parental intentions to vaccinate a subsequent child, and this is a novel finding. Previous studies have shown differences in the perception of vaccines’ safety and efficacy according to vaccination behavior (Bond, Nolan, Pattison, & Carlin, 1998) and that parents of unvaccinated children were less likely to believe in vaccine safety (Salmon et al., 2008; Smith et al., 2011). In a recent qualitative study (Auslander, Meers, Short, Zimet, & Rosenthal, 2019), parents reported that their intentions to vaccinate depended on the benefits and risks associated with these preventive procedures. These parental concerns should be considered in the context of a normative desire to protect their child's health and safety (Bakermans-Kranenburg & Van IJzendoorn, 2017; Schaller, 2018). This emphasizes that parents’ concerns with vaccines’ safety and efficacy should be considered one of the main focuses of preventive interventions.

When deciding on child immunization, many parents may have difficulties understanding the most sophisticated aspects of vaccine research (Kumar et al., 2010). Concomitantly, descriptions of dramatic individual cases, truly or falsely reported in the media or on social networks, can influence parents with lower health literacy levels. Omission bias, in which the harm from action is rated less favorably than the harm from inaction, is a barrier that may be linked to difficulty in processing abstract concepts and probabilistic data (Wroe, Turner, & Owens, 2005).

The answers to the open-ended questions reinforced the results obtained in the survey. Parents with both positive or negative intentions to vaccinate a subsequent child agreed that achieving individual or community immunity is the main reason to vaccinate a child and evoke the fear of negative consequences as a significant motive to withhold vaccines. It is conceivable that the desire to protect and avoid harm for their children triggers the attitude of most parents. However, their different perceptions of efficacy, safety, and understanding of robust research about immunization may lead them into different options.

Most parents with a positive intention to vaccinate showed that they were aware of the motives most commonly invoked to refuse vaccines, suggesting that they had been at least partially exposed to anti-vaccination ideas. However, their responses expressed a rejection of these ideas, mentioning, for instance, that the noncompliant parents are not well informed, ignorant, accept fake news, or hold fundamentalist religious beliefs. These results suggest that exposure to these ideas is not enough to determine parents’ intentions. Some examples included "because they think they are cleverer than all the others" and "because they are selfish." These examples express a refusal of anti-vaccination ideas. However, a few parents showed empathy with nonvaccinating parents ("because indeed the idea of injecting a virus in your child is scary"). These parents might be more susceptible to erroneous information about vaccines or the descriptions of vaccines’ side effects.

Furthermore, approximately half of the parents who intended not to vaccinate their children and answered the open-ended questions were aware of vaccines’ role in achieving individual immunization. This awareness might be insufficient to overcome their fear of the negative consequences of vaccination or their trust in erroneous pseudoscientific information. These parents stated secondary effects, lack of efficacy, beliefs in anti-vaccines conspiracy theory, and lack of information as motives not to vaccinate their children ("because they know vaccines are dangerous," "because nowadays there is a lot of manipulation and vaccines are big business," and "because the organism must gain natural immunity in fighting diseases"). These results point to the need for health professionals to be available and empathically discuss these issues with parents and be particularly attentive to their concerns regarding pediatric vaccines’ safety and efficacy. Even if parents vaccinate their children, they will likely continue to question the need, safety, and efficacy of vaccines, especially if they continue to perceive vaccines as an artificial technology (Reich, 2016).

Considering demographic correlates, parents with more children had a lower intention to vaccinate a subsequent child. Previous studies (Bobo, Gale, Thapa, & Wassilak, 1993; Haynes & Stone, 2004) found that young children from these families showed a higher probability of being incompletely vaccinated. Our results suggest that this lower adherence to immunization schedules might result from both parents’ behavioral barriers and a lower motivation and intention to vaccinate. Other variables not assessed in this study, like religious beliefs, may also affect these results and deserve further exploration.

### Limitations

The results of this study must be considered in the context of its limitations. We only studied the intention to vaccinate a subsequent child, and this dimension may differ from the actual behavior. For some parents who are planning a new pregnancy, this issue may be more critical, and the answers result from a better reflection than for parents who do not have this project. We used a dichotomous measure and, as such, did not capture different levels of hesitancy. We assessed the intention to vaccinate and the related beliefs regarding the recommended vaccines in the national immunization plan in general and did not assess the beliefs concerning specific vaccines. However, although hesitancy differs across different vaccines in several countries, pediatric immunization rates in Portugal are very similar for all NIP vaccines. The study used a cross-sectional design, not allowing the inference of causality. Finally, this was an online study, where invitations reached more educated parents, and probably only highly motivated persons participated. As usual, in most web-based studies, the parents in this sample had a higher level of education than the national population. However, the percentage of parents expressing an intention to vaccinate a next child matched closely with the overall percentage of children complying with the national vaccination plan (DGS, 2019), and all the Portuguese geographical regions were represented.

## Conclusion

Our results showed excellent levels of intention to vaccinate associated with highly favorable beliefs towards pediatric vaccination. When considering all cognitive and sociodemographic dimensions conjointly, the perception of vaccine safety and efficacy was the only significant parental cognitive predictor of the intention to vaccinate a subsequent child, confirming the protection motivation model.

Considering this study's results, we believe that it is essential to develop effective information strategies on vaccines’ safety and efficacy, directed at parents, particularly hesitant parents or those who refused a vaccine in the past, regarding vaccines’ safety and efficacy. As previously stated, our results are consistent with the idea that concerns about pediatric vaccines’ safety might be a significant trigger for parents to refuse to vaccinate their children. The anxiety raised by these concerns may act as a fertile context for anti-vaccination ideas. Previous studies on vaccine hesitancy show that even parents who adhere to national vaccination plans might have many doubts about child immunization (Dubé, Gagnon, Zhou, & Deceuninck, 2016). Our qualitative results suggest that most parents are aware of anti-vaccination arguments, and some of those who intend to vaccinate a subsequent child showed some understanding and empathy for concerns regarding vaccines. Although providers might have trouble handling anti-vaccination ideas, they need to recognize that most parents aim to keep their children safe and healthy. Parents need an empathic and calm provider to help them overcome their anxieties and gain trust in the science behind vaccines and the overall health system.
